# Protein Interaction Network of *Arabidopsis thaliana* Female Gametophyte Development Identifies Novel Proteins and Relations

**DOI:** 10.1371/journal.pone.0049931

**Published:** 2012-12-11

**Authors:** Batool Hosseinpour, Vahid HajiHoseini, Rafieh Kashfi, Esmaeil Ebrahimie, Farhid Hemmatzadeh

**Affiliations:** 1 Institute of Agriculture, Iranian Research Organization for Science and Technology, Tehran, Iran; 2 Department of Biotechnology, College of Science, University of Tehran, Tehran, Iran; 3 Department of Crop Production & Plant Breeding, College of Agriculture, Shiraz University, Shiraz, Iran; 4 School of Molecular and Biomedical Science, The University of Adelaide, Adelaide, South Australia, Australia; 5 School of Animal and Veterinary Sciences, The University of Adelaide, Adelaide, South Australia, Australia; University of Jaén, Spain

## Abstract

Although the female gametophyte in angiosperms consists of just seven cells, it has a complex biological network. In this study, female gametophyte microarray data from *Arabidopsis thaliana* were integrated into the *Arabidopsis* interactome database to generate a putative interaction map of the female gametophyte development including proteome map based on biological processes and molecular functions of proteins. Biological and functional groups as well as topological characteristics of the network were investigated by analyzing phytohormones, plant defense, cell death, transporters, regulatory factors, and hydrolases. This approach led to the prediction of critical members and bottlenecks of the network. Seventy-four and 24 upregulated genes as well as 171 and 3 downregulated genes were identified in subtracted networks based on biological processes and molecular function respectively, including novel genes such as the pathogenesis-related protein 4, ER type Ca^2+^ ATPase 3, dihydroflavonol reductase, and ATP disulfate isomerase. Biologically important relationships between genes, critical nodes, and new essential proteins such as AT1G26830, AT5G20850, CYP74A, AT1G42396, PR4 and MEA were found in the interactome's network. The positions of novel genes, both upregulated and downregulated, and their relationships with biological pathways, in particular phytohormones, were highlighted in this study.

## Introduction

The plant life cycle alternates between a haploid gametophyte and a diploid sporophyte generation. Flowering plants have two gametophytes: the female gametophyte (FG) and the male gametophyte. The FG (embryo sac) most commonly consists of seven cells and four different cell types, including one egg cell, one central cell, two synergid cells and three antipodal cells. The FG cells control many steps of the fertilization process. During pollen tube growth, the synergid cells produce a guidance cue that directs pollen tube growth to the ovule [1].

Following fertilization, the egg cell gives rise to the seed's embryo, and the central cell develops into the endosperm. Differentiation and development aspects of this tiny organ have been studied by many groups, resulting in the identification of many specific genes that are necessary for FG development. Several expression-based studies have complemented forward genetics approaches which led to the identification of hundreds, but not all of the FG-expressed genes [2], [3], [4], [5], [6].

Additionally, there are some efforts to understand how the FG derives its specificity; which genes are expressed differentially in the embryo sac cell types and how these genes are regulated. A few of these genes have been analyzed functionally, such as *FERTILIZATION-INDEPENDENT ENDOSPERM (FIE)* [7], *MEDEA (MEA)* [8] and *AGAMOUS-LIKE* 80 *(AGL80)* [9].

There is no report, however, about the interaction networks that underlie these biological processes. It should be noted that proteins have many important roles, not only at the single cell level but also at the tissue level. They act as catalysts, structural components, messengers, regulators and interact in a very concerted network of different pathways. The protein interaction (PI) network helps researchers to better understand these biological systems.

PI networks can be constructed using both experimental and computational methods. Experimental methods, such as yeast two-hybrid and tandem affinity purification, are reliable methods to extract direct interactions, but these methods are difficult to use on a large scale, which results in incomplete networks and a limited number of discovered interactions [10]. In fact, these methods also suffer from the low coverage of protein interactions. In addition, a complete interactome for many organisms is not available. Therefore, computational methods play pivotal role to predict interactions using available biological data.

Several computational methods have been developed to predict dynamic PI networks based on their action at the molecular level (molecular function) or their performance at the cellular level (biological process) including co-evolution pattern, co-expression, co-occurrence in interacting proteins, protein domains and phenotypes, sequence and protein structure [11]. Co-occurrence hypothesis assumes that some proteins with related functions are co-regulated as gene clusters or presented in unit-like operons. The main idea in co-evolution based methods is that functionally linked proteins have correlated evolution changes and mutual selective pressure. In co-expression method, the hypothesis proposes that a protein complex is composed of functional subunits, and consequently, expression levels of all subunits should be correlated. To predict protein-protein interactions, co-expression method searches for the relative expression levels of subunits in gene expression data and finds significant correlation between them [12]. Large amounts of PI data have been generated by both computational and experimental methods. These data are stored and are available for biological researches. Two of most comprehensive interaction databases are the Biomolecular Interaction Network Database (BIND) [13] and the Database of Interacting Proteins (DIP) [14].

Studies aimed at elucidating the PI networks, involved in different human biological processes, have been carried out by integrating available transcription and interaction data [15], [16], [17]. By exploiting this method, He *et al.* (2010) [18] constructed a dynamic protein-protein interaction network for the floral transition process in *Arabidopsis*. In addition, Yu *et al.* (2008) constructed a chloroplast PI network based on literature-mined data and computational methods [19]. Since genome sequences of many organisms are now available, different groups have tried to predict PI networks of them using computational methods. The predicted interactions are integrated into softwares such as Pathway Studio (Ariadne Genomics, Rockville, MD). Besides protein-protein interactions, Pathway Studio enables us to understand other interaction types of proteins with small molecules, their relations with biological processes and molecular function of PIs [20]. Indeed, experimental based data can be used to draw out more specific and reliable PI networks involved in different biological mechanisms of an organism.

This work is the first attempt to construct *Arabidopsis thaliana* FG developmental PI networks based on the molecular functions and biological processes of proteins by integrating computational and experimental based data. The main strategy was to select upregulated and downregulated genes using available microarray data. Then the data were employed to construct PI network, based on shortest path algorithm by Pathway Studio software. In shortest path method, shortest paths between selected protein and any protein in the biological group can be found. Consequently, this method finds the closest protein in biological group regulating genes in FG development.

PathwayStudio is a pathway analysis tool supplied with RESNET database. PathwayStudio harvests latest information from deposited literature in PubMed and other public sources. The software also uses a number of public and commercial databases such as KEGG (metabolic database; http://www.genome.jp/kegg/), BIND (protein interaction database; http://www.bind.ca), and GO (Gene Ontology) [20]. RESNET product includes database of relations for mammalians and plants as well as molecular network databases for model organisms such as plant model, *Arabidopsis thaliana*. Plant RESNET contains protein-protein relations which include protein-protein binding, expression regulation, promoter binding, protein modification, effects of different proteins and environmental conditions on small molecule and metabolite synthesis, molecular synthesis relations, regulation of proteins by plant hormones, and unknown regulation from small molecule to a protein.

In next step, we carried out a deep analysis on both structural and biological aspects of the constructed network to provide comprehensive information on different interactions which may happen in the cell such as protein-protein interactions, protein complexes, small molecule synthesis, and expression regulation. Furthermore, since a network is composed of subnetworks, we extracted small subnetworks of some specific proteins to find the possible relations with other subnetworks.

In addition, we sought for essential proteins based on topological analysis of the networks. Several algorithms were designed to find essential nodes/hubs in the networks. The bottleneck (BN) and double screening scheme (DSS) methods were used in our analysis. Bottleneck nodes are defined using topological feature “betweenness” [21]. It is the total number of nonredundant shortest paths going through a certain node or edge [22], [23]. Nodes with the high betweenness are major intersections between clusters. If bottlenecks are removed, a network can be partitioned and biologically they are usually essential proteins. Idowu *et al.* (2004) [24] used degree and Bottleneck methods to identify the possible-essential proteins in the PI network of *Bacillus subtilis*.

DSS employs two methods for scoring: Maximum Neighborhood Component (MNC) and Density of Maximum Neighborhood Component (DMNC). Lin *et al.* (2008) [25] applied different scoring algorithms to analyze yeast PI dataset and evaluate the performance of all algorithms by the coverage of the essential proteins. Interestingly, in their study, 80% and 60% of the top 10 proteins found by DMNC and MNC have been identified as yeast essential proteins by previous experimental methods. However, they integrated DMNC and MNC methods as DSS and found that DSS improves hit rates up to 100%. After DSS, BN method has the high hit rate (60%) on the essential protein list.

In this investigation, novel upregulated and downregulated genes were identified in the networks and their predicted functions were discussed. Interestingly, novel genes were identified as critical/essential proteins which had not been reported previously. Furthermore, we uncovered relations of some important proteins with other proteins, molecules, and biological processes. The present analysis is the first study in prediction of PI network in *Arabidopsis* FG development and provides required knowledge and view for future studies.

## Materials and Methods

### Microarray datasets

The *Arabidopsis* FG protein interaction network was inferred by using the upregulated and downregulated genes that have been previously shown to be expressed in the FG. We used the *Arabidopsis* FG Affymetrix ATH1 oligonucleotide microarray data published by Yu *et al.* (2005) [4] (available online at www.plantphysiology.org) and Steffen *et al.*(2007) [3]. Yu *et al.* (2005) [4] identified genes involved in female gametophyte development by comparative gene expression profiling between wild-type ovules and *spolocyteless (spl)* mutant ovules. Steffen *et al.* (2007) [3] also applied the same method for gene identification in *Arabidopsis* FG using *male sterility* 1 (*ms*1) as the source of normal ovules and *determinant infertile* 1(*dif*1) as a source of mutant ovules.

Regarding the aim of the study on prediction of protein interaction map, we screened genes with a fold change greater than 1.5 and *p*-values less than 0.05 as upregulated candidates, and genes with a fold change less than 0.66 and *p*-values less than 0.05 as downregulated candidates. Genes identified by Wang *et al.* (2010) [26] using real-time PCR were also added (see Table S1 for a list of these genes).

### Construction of the PI networks

To understand the system-level dynamics of a living organism, information about protein function and cellular pathways is needed. Much of this information is scattered throughout numerous scientific publications. Software systems can bring the relevant information together, organize and prepare the information for further analysis.

To construct our PI network, Pathway Studio software (Ariadne Genomics, Rockville, MD) was used. Pathway Studio uses the RESNET Plant database, which is a comprehensive pathway and molecular interaction database in plant science. This database includes new aliases for *Arabidopsis* genes and new entries from six other plants, including rice, barely, corn, tomato, potato and tobacco. The database also includes other data directories, such as PubMed and TAIR. Additionally, it has information on functional relationships and molecular interactions that have been obtained from the literature.

The software collects information using text-mining tool MedScan and process undertaken data by natural language processing (NLP). The language interprets them to logical concepts and extracts functional relationships between proteins, small molecules, and cellular processes. The software is equipped with several layout algorithms for drawing links and visualization of the network [20].

In an interaction network, each node is a protein or small molecule, and the nodes are connected to each other by edges. Connectivity, or degree, is the number of edges linked to a single node, which can be interpreted as the number of interacting partners of a given protein. The shortest paths between a given entity and its group were selected to construct the networks [27]. The shortest path length is the path with the smallest number of links between a pair of nodes [28].

The software defines two types of entities: simple or complex. Simple entities including proteins or small molecules have no components themselves and are atomic objects. Simple entities may be part of a network. By contrast, complex entities may also be part of a pathway. They differ from simple entities in that they may contain proteins involved in cell processes, functional classes such as enzyme families and protein complexes (a group of two or more proteins linked by non-covalent protein-protein interactions). To predict protein interactions, the software makes different groups of proteins and finds a relation between a protein and its group. So a biological group is container of one or more simple or complex entities and used for enrichment analysis using algorithms such as Fischer's Exact Test. Proteins in the groups can be moved to a pathway based on found relationships.

Networks were constructed based on molecular functions (to draw out clusters based on their related function) and biological processes with defined genes. The excel format of the networks are provided at the corresponding supplementary files. Each excel file of the networks has all relations and entities of the network. Non-directed links and arrows represent PIs and regulatory effect of interactions, respectively. Left- right arrow shows reciprocal interaction. Effects can be activation (+) or downregulation (−) forms. PI modes of proteins include binding (physical interaction), protein modification of target molecule, direct regulation of target by physical interacting the protein with it, expression (changing the protein level of the target), promoter binding, molecular synthesis (changing the concentration of the small molecule by protein), molecular transport (protein changes the localization of the target), regulation (protein changes the activity of the target), and chemical reaction (protein works in e.g. enzyme catalyzing reactions). Additionally, the references of relations coming from literature are provided in “MedlineReference” column.

**Figure 1:**
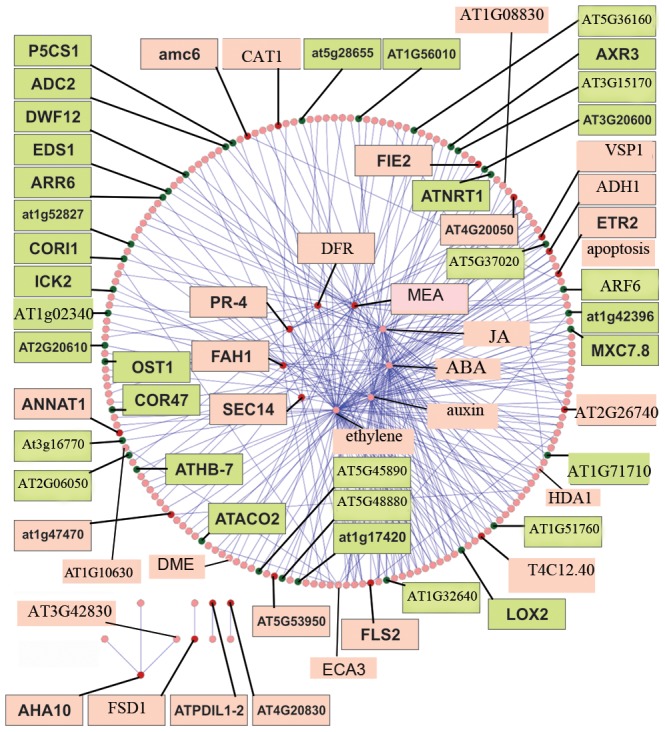
Subtracted interaction network of some nodes based on biological processes. MEA, DFR, JA, ABA, auxin, ethylene, SEC14, FAH and PR-4 formed an interconnected sub-network, while AHA10, FSD1, ATPDIL1-2 and AT4G20830 formed a separate, small sub-network. The network was visualized using Cytoscape software. Upregulated proteins are colored red, and downregulated proteins are colored green. The Excel file of the map can be found in Table S4.

To analyze in depth, we subtracted (reduced) the primary network based on the biological processes into smaller networks or subnetworks. As a result, a subnetwork is a part of primary network with more focus on biological processes. MATLAB programming language was used for subtraction. Using microarray output data, upregulated and downregulated genes were selected in the primary network, and subnetworks containing these genes were extracted. At the extracted subnetwork, at least one of the two nodes in each interaction had significant expression level. The network was visualized by Cytoscape software (Fig. **1**).

**Figure 2:**
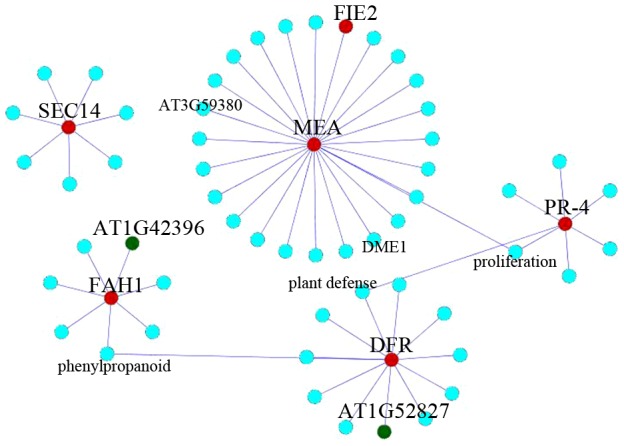
Subtracted interaction network of several upregulated FG proteins based on biological processes. Relations between hubs of MEA, FAH1, PR-4, SEC14 and DFR are shown. upregulated and downregulated genes are shown in red and green respectively. Relations were extracted from the primary network using a language program “MATLAB”. Using language program, it was asked for all the interactions between MEA, SEC14, PR-4, DFR and FAH1 from network to predict which proteins or biological processes are affected by their interactions. As shown, MEA and PR-4 are linked to each other by proliferation process. FAH1 (involved in cell death program) is linked to plant defense through DFR and phenylpropanoid. But SEC14 was not connected to any of other four proteins.

We also extracted subnetworks of several proteins including MEA, SEC14, FAH1, PR-4, and At5g42800 as at least one of the two nodes in each interaction had significant expression level. The interesting relations between subnetworks were visualized by Cytoscape software (Fig. **2**).

### Essential proteins/hubs analysis

Cyto-Hubba, as a hub finder/analyzer software, was used to find essential proteins/hubs [25]. These essential proteins/hubs may serve as candidates of novel genes involved in FG development. The subnetworks of these essential nodes provide the opportunity of a more precise understanding of the biological functions providing valuable clues for biologists. Cyto-Hubba is a Java plugin for the Cytoscape software enabling us to find critical nodes or hubs and fragile motifs in an interactome network by several topological algorithms, including Degree, BN, Edge Percolated Component (EPC), MNC, DMNC, and a DSS of MNC and DMNC [25], [29], [30]. As mentioned above, we used BN and DSS to find the top ranked essential proteins in the networks based on biological processes and molecular function. To do this end, all relations in unfiltered network were transferred to Cytoscape software and analyzed by Cyto-Hubba plugin.

### BN method

For each node v in an interaction network, a tree of shortest paths starting from v is constructed, called BN. Taking v as the root of the tree Tv, the weight of a node w in the tree Tv is the number of descendants of w, that is to say, equal to the number of shortest paths starting from v passing through w. A node w is called a bottle-neck node in Tv if the weight of w is no less than n/4, where n is the number of nodes in Tv. The score of node w, BN(v), is defined to be the number of node v such that w is a bottle-neck node in Tv.

### DSS method

Each scoring method catches a certain topological characteristic of essential proteins. But in DSS, two scoring methods DMNC and MNC, are used to extract mixed characteristic of the essential proteins. For n, most possible essential proteins are expected in the output, the 2n top ranked proteins by method DMNC are selected firstly. The selected 2n proteins are further ranked by method MNC and the n top ranked proteins are outputs. The number 2n is an empirical value for this double screening method.

Finally, we verified our work using some of the important proteins that were identified by cyto-Hubba based on previously-defined genes by Yu *et al.* (2005) [4], Steffen *et al.* (2007) [3], Wuest *et al.* (2010) [5], and Johnston *et al.* (2007) [6].

### Gene Ontology analysis

A cytoscape plugin, the Biological Networks Gene Ontology tool (BiNGO 2.3) was used to identify overrepresented gene ontology (GO) terms. Over-represented GO terms were selected using corrected *p*-values at significance levels of *p*<5E-5 and *p*<0.05.

## Results

### PI network based on biological processes

The primary PI network based on biological processes is presented at the Table S2 in excel format. Due to the large size of the primary network, it was subtracted to a smaller network as described in materials and methods section. Subtracted network was composed of 632 nodes and 2362 edges (Table S3). The average degree (connectivity) of the network was seven. The nodes include 245 previously-identified genes (74 upregulated and 171 downregulated genes) (Table S3). Fifty-one out of 245 genes were transcription factors (10 were upregulated and 41 were downregulated). There were also 12 transporters, including 4 from the upregulated set and 7 from the downregulated set. Due to the large size of the network map, we created an image of the subtracted network based on some of the most previously known proteins, which is shown in Figure 1 and Table S4.

The network also comprises important functional groups, such as hormones, plant defense proteins, polycomb group (Pc) proteins, signal transduction proteins and MADS box proteins, which have high numbers of connections. There are some main functional groups, including apoptosis, gametophyte development, cell division, and methylation, with fewer connections (Table S3). This can be due to the lack of information in the latter cases compared to the former groups. CYP74A, an allene oxide synthase with 117 connections, had the highest number of connections in the network. CYP74A is downregulated in FG according to microarray analysis [4]. Alcohol dehydrogenase 1 (ADH1) has the highest connectivity number of the upregulated genes (75 connections).

The global view of the network reveals several main groups connected to previously-indentified genes or genes with high and low fold changes (Table S3). These groups are illustrated below.

### Plant defense-related proteins

Proteins involved in plant defense and response to disease and pathogens formed main hubs in the network (Table S3). For instance, PR-4 (Pathogenesis-related Protein; At3G04720) forms an interesting sub-network, which suggests versatile roles for this protein (Fig. **2**). It has link to plant defense proteins' node and also interacts with proteins involved in the ethylene pathway, cell growth, and proliferation. PR-4 has reciprocal interactions with the salicylate and ethylene signal transduction pathways.

### Polycomb Group

The Pc group proteins are involved in distinct developmental processes, such as seed development and floral induction. One of the Pc group members, MEA, is observed in the network (Fig. **1** and Table S4). MEA is linked to other Pc group members, including FIE, FIS2 and DME1 (Fig. **1**). Additionally, it is linked to some subnetworks that are represented in the subtracted network of MEA (Fig. **2**). The subnetworks comprising MEA, PR-4, At5G42800 and FAH1 (a fatty acid hydroxylase) were interconnected. MEA also has connections to AT3G59380 (a farnesyl transtransferase) and ORC2 (Origin recognition complex subunit 2). AT3G59380 is involved in protein prenylation [31].

### Anthocyanin biosynthesis

Anthocyanin is one of the flavonoids in plants. In our network, anthocyanin formed a distinct sub-network that was linked to upregulated and downregulated genes inside various biological groups, such as glutathione S-transferase 26, chalcone synthase (CHS), AT5G42800, tannin deficient seed 4, JA (Jasmonic acid), and banyuls (it is a mutation in AT1G61720 which causes a color similar to local wine “banyuls” in seed coat) (Table S3). AT5G42800, which is also known as dihydroflavonol reductase (DFR), is involved in the flavonoid biosynthesis pathway and has a hub in the network (Table S3). DFR also interacts with the downregulated protein AT1G52827, cadmium tolerance 1 and JA. Figure **2** shows the subtracted interaction network of this protein. Furthermore, the DFR hub interacts with the FAH1 and PR-4 sub-networks. FAH1 is involved in programmed cell death [32].

### Signal transduction

There were signal transduction and signaling pathway nodes in the network with interesting links to phytohormones and plant defense groups (Table S3). For example, flagellin-sensitive 2 (FLS2) was linked to plant defense proteins and signal transduction regulatory subnetworks. Ethylene Response 2 transcription factor (ETR2) was connected to the regulation of signal transduction, abscisic acid (ABA), and ethylene subnetworks. Upregulated ADH1 contributed to the ethylene pathway and regulation of signal transduction, and ABA positively interacted with ADH1 (Fig. 1). Additionally, EDS1 (a signal inducer, Fig. 1), AT3G20600 (non race-specific disease resistance 1, Fig. 1), CYP74A, AT2G30040 (a kinase, Table S3), AT4G21120 (CAT1, a cationic amino acid transporter, Fig. 1) and ethylene were linked to the regulation of signal transduction.

JA plays roles in signaling pathways and interestingly, it was present in the network. JA node interacts with two upregulated genes: AT5G42800 and ATVSP1 (Table S3). Downregulated Histidine-containing phosphotransmitter2 (AHP2) and EOSTRE (a BELL-like homodomain protein) were also observed in the network (Table S3).

### Transporters

There were several different transporters in the network, including SEC14 (a phosphatidylinositol transporter), electron transporters (AT4G20830), nucleoside transporters, CAT1 and ion transporters. Regarding propensity of transporters in the network, we further analyzed the different transporters represented in the network (Fig. **1** and Table S3). The sub-network of SEC14 is represented in Figure 2.

Sucrose-proton symporter 2 (ATSUC2) had a high number of connections in the network (Table S3). ATSUC2 is involved in loading sugar from the apoplast to conductive elements [33]. F14P1.2, AT1G19650, a Sec14p-like phosphatidylinositol transfer family protein, is linked to the upregulated AT1G22530, a patellin 2 transporter (Table S3). Additionally, the downregulated protein F14O23.9, AT1G71710, is connected to the phosphatidylinositol node (Fig. 1, Table S3). AT1G71710, is a member of the DNase I-like superfamily. The upregulated protein Autoinhibited H^(+)^-ATPase isoform 10 (AHA10) has constructed a small sub-network that is separated from the core network (Fig. 1, Table S3). AHA10 belongs to the H^+^-ATPase gene family and is involved in ion transportation. It is upregulated in FG and interacts with the zinc finger protein AT3G42830.

### Phytohormones

Presence of different phytohormones in the network is interesting (Fig. **1**). The ethylene pathway had the greatest number of connections including gibberellin, ABA, and auxin. All of these hormones were linked to the signal transduction pathway.

Auxin showed an interaction with the node made up of the upregulated proteins FLS2, AT2G26740 (an epoxide hydrolase), AT5G53950 (CUC2), and JA (Fig. 1). AT2G26740 encodes a soluble epoxide hydrolase that is connected to the auxin node. Additionally, Figure 1 shows that Auxin Response Factor 8 (AT5G37020) is also present in the network. ARFs can bind to auxin response promoter elements and regulate gene expression in response to auxin [34].

It is apparent from Figure 1 that ABA is linked to some of the upregulated genes, including ADH1 and AT4G21120 and also, to some of the downregulated genes, such as dehydrin protein (COR47) and Athomobox 7 (ATHB7). The plant defense node was connected to the ethylene and ABA pathways (Table S3).

The ethylene pathway is predicted to have a positive effect on differentiation and histone deacetylase 1 (HDA1) expression (Fig. 1). Additionally, among phytohormones, the apoptosis pathway had a direct interaction only with ethylene (Fig. 1).

Another interesting finding was that only downregulated genes, such as SUPERMAN, SHI, T25B24.2 (a nucleoside transporter) and Reduced Sensitivity to Far-Red Light 1 (AT1G02340, a bHLH transcription factor) were linked to the GA node (Table S3).

### Miscellaneous nodes/subnetworks in the network based on biological processes

Further analysis of the network indicated other interesting nodes and interactions. For example, AT5G43035 (calmodulin-binding protein, Table S3), ANNAT1 (Fig. 1) and ANNAT4 (calcium binding proteins) were seen in the network (Table S3). The copper ion binding protein, AT4G39830, was upregulated in FG interacting with the downregulated gene AT1G24470, which is a beta-ketoacyl reductase (Table S3).

The apoptosis node had links to the upregulated gene AMC6 (Fig. 1) and the downregulated gene SPL11 (AT1G27360) (Table S3). Interestingly, FAH1 (AT2G34770), a negative regulator of programmed cell death, contains a hub in the network (Fig. **1** and **2**).

Another interesting node was ECA3, a ER-type Ca^2+^-ATPase 3 protein (Fig. 1, Table S3). ECA3 encodes a Golgi-localized P2A-type Ca^2+^ ATPase. As shown in Figure 1, ECA3 interacts with AT1G42396 (a downregulated sterol methyltransferase 1), AT1G01910 (a putative anion-transporting ATPase), AT5G20850 (ATRAD51), ADP-ribosylation factor A1F (AT1G10630) and the upregulated superoxide dismutase (AT1G08830). ECA3 is involved in calcium and manganese transportation [35].

The glutathione node is connected to the upregulated gene, GGT1 (AT4G39640), a gamma-glutamyltransferase (Table S3). There are four GGTs in the *Arabidopsis* genome [36]. Therefore, we searched for three additional GGTs. Interestingly, GGT2 (AT4G39650) was expressed in FG at a level similar to that of GGT1, whereas GGT3 and GGT4 were downregulated (Table S3).

Upregulated MYB98 (a member of the R2R3-MYB gene family), downregulated Histidine-containing phosphotransmitter2 (AHP2) and EOSTRE (a BELL-like homodomain protein) were observed in the network (Table S3). The DD39 (At4G20050) node in the network is connected to pollen development (Fig. **1**). DD39 is strongly expressed in anthers, but not in pollens. It was very interestingly that DD39 has interaction with ECA3 as shown in Figure 1.

### PI network based on molecular function

**Figure 3:**
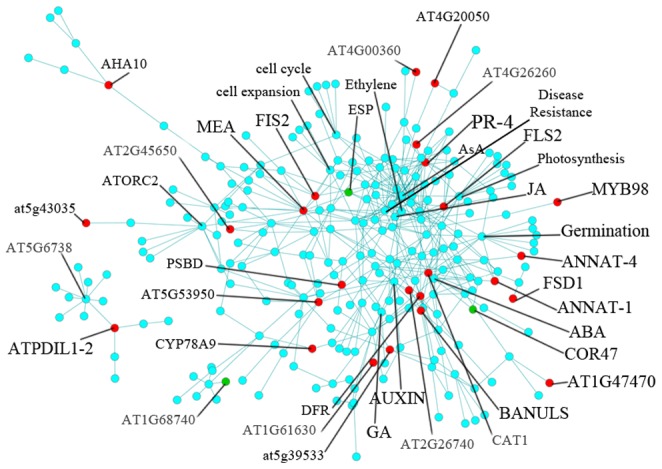
Interaction network of FG proteins based on molecular function. The network was visualized using Cytoscape software. upregulated proteins are indicated in red, and those that are downregulated are shown in green. Excel file of the map have been provided in Table S5.

Using Pathway Studio, we constructed the functional organization of the interaction network. The total network consisted of 260 nodes and 453 edges, 24 of which were previously-defined as upregulated genes and 3 of which were downregulated genes in FG (Fig. 3 and Table S5).

There were different functional groups in the network, which had the same trend with the biological process based network (Fig. 3). Ascorbic acid and plant defense nodes had the highest number of edges (25 and 19, respectively).

PR-4 showed connections to several functional groups, such as the transduction and plant defense nodes. Fe-superoxide dismutase (FSD1), one of the upregulated genes in the network, is linked to the saline node (Fig. 3). Auxin shows interactions with three upregulated genes: AT5G53950, FLS2 and AT2G26740, similar to the biological process based network.

The JA node was linked to one upregulated gene, DFR. The pathogenesis node had an interaction with the upregulated unknown gene AT1g47470. AT4G21120 was in the network and had a degree of 12. CAT1 interacts with the regulation of signal transduction and ABA response nodes.

ATPDIL1-2, an electron transporter, formed a separate, small sub-network. It is a member of the protein disulfate isomerases (PDIs). ATPDIL1-2 is upregulated in FG. This protein was predicted to bind AT5G67380 ( a Casein kinase II).

### Over-represented GO terms

Using a Cytoscape plugin, BiNGO, we assessed whether there are any over-representation of Gene Ontology categories in the networks [37]. Significantly over-represented GO terms included those that are related to cellular processes, responses to different stresses, plant defense, RNA processing (such as Suppressor-of-White-Apricot proteins), cell cycle, cell fate specification, polarity specification cell plate formation, protein kinase activity and signal transduction in the biological process based network (Table 1, Table S6). However in the network based on molecular functions, some significantly large overrepresented GO terms were related to catalytic activity, hydrolase activity, DNA binding and transcription activator activity (Table 2, Table S7).

**Table 1:** Over-represented GO terms (“biological process” category) in the FG interaction network.

GO-ID	Corrected p-value	Number of annotations	Description
51869	7.17E-39	114	response to stimulus
9628	3.05E-27	56	response to abiotic stimulus
6950	2.37E-26	75	response to stress
42221	2.37E-26	64	response to chemical stimulus
32501	1.04E-23	62	multicellular organismal process
32502	3.30E-23	64	developmental process
48856	8.39E-22	52	anatomical structure development
9719	1.44E-21	45	response to endogenous stimulus
7275	2.38E-19	55	multicellular organismal development
9725	2.36E-16	37	response to hormone stimulus
50876	3.51E-16	39	reproduction
8151	5.60E-16	164	cellular process
22414	1.12E-15	38	reproductive process
3006	1.12E-15	35	reproductive developmental process
48608	1.12E-15	35	reproductive structure development
9605	1.45E-14	23	response to external stimulus
6970	2.94E-14	22	response to osmotic stress
48731	8.08E-14	30	system development
48513	8.08E-14	30	organ development
9791	3.89E-13	24	post-embryonic development
65007	8.68E-13	79	biological regulation
50793	9.71E-13	21	regulation of developmental process
51706	8.17E-11	27	multi-organism process
6349	1.29E-10	7	genetic imprinting
9737	1.86E-10	17	response to abscisic acid stimulus
9651	2.45E-09	16	response to salt stress
9314	2.49E-09	21	response to radiation
48519	2.49E-09	16	negative regulation of biological process
48409	2.49E-09	15	flower development
50791	5.20E-09	67	regulation of biological process
19748	1.25E-08	21	secondary metabolic process
9414	1.57E-08	13	response to water deprivation
51707	1.62E-08	21	response to other organism
9887	1.64E-08	14	organ morphogenesis
9607	3.11E-08	22	response to biotic stimulus
6394	3.92E-02	9	RNA processing

Corrected p-values less than 5 E-5 were considered statistically significant.

An Excel file of all GO terms is available in Table S6.

**Table 2:** Over-represented GO terms (“molecular function” category) in the FG interaction network.

GO-ID	Corrected p-value	Number of annotations	Description
4302	1.30E-05	42	carboxylesterase activity
30599	2.10E-05	21	pectinesterase activity
4857	2.10E-05	27	enzyme inhibitor activity
16787	3.55E-05	213	hydrolase activity
46910	1.44E-04	16	pectinesterase inhibitor activity
30234	1.44E-04	36	enzyme regulator activity
16563	5.42E-04	17	transcription activator activity
16798	8.37E-04	48	hydrolase activity, acting on glycosyl bonds
4553	1.04E-03	44	hydrolase activity, hydrolyzing O-glycosyl compounds
46423	1.25E-03	4	allene-oxide cyclase activity
3883	5.38E-03	4	CTP synthase activity
3824	5.70E-03	502	catalytic activity
16813	1.41E-02	3	hydrolase activity, acting on carbon-nitrogen (but not peptide) bonds in linear amidines
16788	2.09E-02	75	hydrolase activity, acting on ester bonds
4650	2.78E-02	13	polygalacturonase activity
5507	3.86E-02	17	copper ion binding
8526	3.86E-02	3	phosphatidylinositol transporter activity
8497	3.86E-02	3	phospholipid transporter activity

A corrected p-value less than 5E-2 was considered statistically significant. An Excel file of all GO terms is available in Table S7.

### Essential proteins/hubs in each of the networks

**Figure 4:**
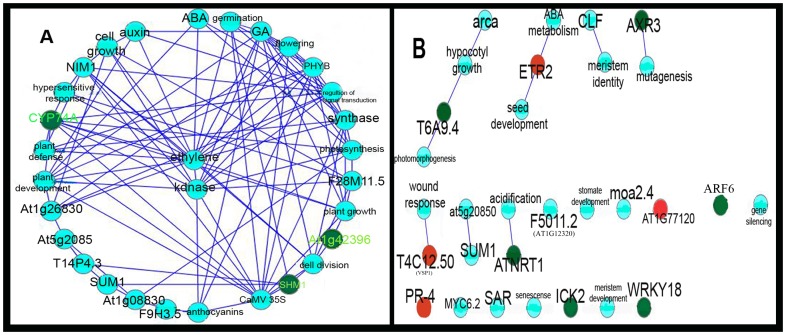
Identification of essential proteins/hubs in the biological process network using BN and DSS method. Figures A and B show the top 30 highest ranked hubs identified by BN and DSS methods, respectively. AT1G26830, AT5G20850, CYP74A, AT1G42396, MEA and SHM1 were identified using the BN algorithm, and AT1G02340, ETR2, VSP1 and PR-4 were identified as critical controllers using the DSS algorithm. upregulated genes are colored red and downregulated genes are colored green.

Using the Hub Objects Analyzer (Hubba) software, key controllers in the networks were identified based on the Bottleneck (BN) algorithm and the double screening scheme (DSS). The top 30 highest-ranked hubs were predicted in the network based on biological processes. AT1G26830 (Cullin), AT5G20850 (DNA repair RAD51/transcription factor, CYP74A, AT1G42396 (hypothetical protein), MEA, SHM1 (serine hydroxymethyl transferase), GA, auxin, ABA and ethylene were some of the critical controllers identified by BN method (Fig. **4A**); however, AT1G02340, ETR2, ADH1, VSP1 (an acid phosphatase) and PR-4 were examples of the essential proteins identified using the DSS method (Fig. **4B**) in the network based on the biological processes.

**Figure 5:**
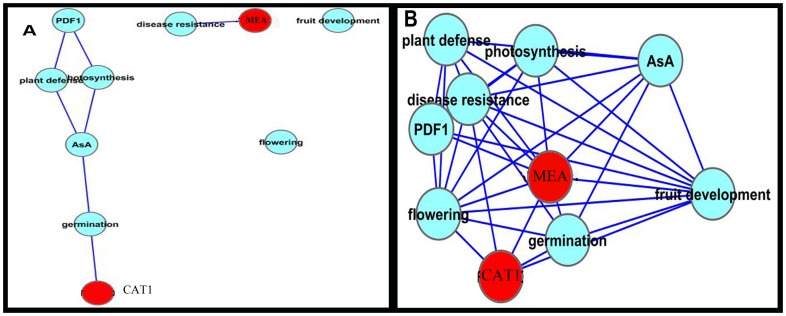
Identification of essential proteins/hubs in the molecular function network using the BN and DSS methods. Figures A and B show the top 10 highest ranked hubs identified by BN and DSS methods, respectively. Red proteins are upregulated based on the microarray data.

The top 10 highest ranking hubs identified by the BN and DSS methods are shown in Figures 5A and 5B for molecular function based network. In the network based on molecular functions, DFR, MEA and CAT1 were among proteins that formed critical nodes.

## Discussion

The present study was designed to determine the first PI network of FG development in *Arabidopsis* by integrating interactome and transcriptome data. Although different groups have studied FG transcriptome using various experimental methods such as forward genetics or microarray technique and produced informative expression data, but none of previous studies has investigated FG protein interaction network [2], [3], [4], [5], [6]. Here in this study, PIs based on biological processes and small molecules were analyzed. Due to complexity of FG development which has been previously indicated by other researcher groups [5], [6], PI network offers more visualize and understandable approach in analyzing FG development. Furthermore, this network can predict undiscovered proteins in FG development and highlight key proteins based on network topology.

To construct PI network, we obtained microarray output data produced by Yu *et al.* (2005) [4] and Steffen *et al*. (2007) [3]. Upregulated genes as well as downregulated genes were selected and used to construct the PI network. An implication of this is the possibility of interactions between upregulated and downregulated proteins. We also hypothesized that downregulated proteins may help us to find bottleneck proteins or easily to say “bridges” between an activated and inactivated processes. On the other hand, by including downregulated proteins, the probability of a bias in the network toward the upregulated proteins reduces. There are potential biases in large interaction networks because of incomplete coverage of the networks and high number of edges for most studied proteins but interaction network is still the first choice for deciphering complex biological systems [38].

For construction of the networks, the shortest path method was used. The method identifies the shortest path length between two nodes to connect them [27]. This approach has been previously validated for construction of biological networks [27]. They found that the shortest path method is a useful method for construction of longevity network. Also, they demonstrated that this method can reduce the size of the network and increase the possibility of the positive interactions compared to other methods.

Various pathways were observed in the network, such as reproduction, hormone-mediated signaling, response to stress, developmental process, signal transduction, seed development, flowering, pollen development and defense response. Previous functional studies indicated that different biological processes such as plant defense, regulation, signaling, reproduction and phytohormones are involved in FG development [4], [5], [6]. PI network of such complex tiny organ would be considerably large. Therefore, we extracted subnetworks of previously identified up- and downregulated proteins from our constructed networks and analyzed their relations. Interconnections of these biological processes combined with the expression patterns of proteins provided the opportunity of analyzing the specification mechanism of different cell types of FG.

In the network based on the biological processes, PR-4 had interaction with salicylate and ethylene signal transduction pathways which is consistent with the findings of Glarebrook *et al.* (2003) [39] and Norman-Setterblad *et al.* (2000) [40]. These investigations showed that PR-4 expression is induced in the JA and ethylene pathways. PR-4 also had a direct link to plant defense proteins. PR-4 also was found as critical node in the network (Fig. 3B and 4B). Several plant defense proteins have been identified with significant fold changes using microarray experiments [3], [4]. Steffen *et al.* (2007) [3] identified several defensin-like proteins specific to synergid and central cells (DD2, DD25 and DD37). Defensin-like proteins are thought to have roles in plant disease resistance; however, they may also have other important roles in plant development. The maize defensin-like protein ZmES4 is active in synergid cells and is required for pollen tube burst. This protein is active on the potassium channel KZM1 as well [41].

The Pc proteins have an important role in epigenetic control of development. MEA, one of the Pc proteins, was present as a bottleneck protein in the network (Fig. 2). Mutations in MEA cause endosperm development in the absence of fertilization [8]. MEA showed an interesting subnetwork connected to PR-4, DFR and FAH1 subnetworks. MEA had connection to AT3G59380 with prenylation activity. This function is necessary for many proteins involved in cell division, growth and signaling. Prenylation facilitates membrane association and protein–protein interactions. Mutation in AT3G59380 produces self-fertile flowers and fewer seeds in *Arabidopsis* [31]. Regarding to MEA and ORC2 relation, it was previously found that ORC2 is expressed in central cell and synergids [42]. Various interactions of MEA with proteins involved in different biological processes such as proliferation, programmed cell death and plant defense are implications for being as a bottleneck protein.

Anthocyanin, a class of chemicals, plays a role in the pigmentation of fruits and flowers, developmental processes, and stress tolerance. DFR is one of the seven different flavonoid enzymes in *Arabidopsis* that is a single-copy gene [43]. It is interacted with PR-4 and FAH-1 in the network.

The signaling pathways involved in FG development are not well understood, but these pathways are likely to be crucial for linking different processes during growth, differentiation, specification, and crosstalk between different cell types in the embryo sac and pollen [44]. The role of JA in signaling pathways has been extensively studied, and JA was shown to have important roles in reproductive processes [45]. In addition, some JA mutants in tomatoes were shown to have a sterile female phenotype [46].

Although transporters have been studied in relation to biotic and abiotic processes, little is known about their function in FG development. As an important node in the network, SEC14 is a major phospholipid in eukaryotic cells and is considered to be critical in different developmental and cellular processes. Various phosphatidylinositol-regulated genes are expressed specifically during different environmental conditions [47]. AtSUC1 and ATPDIL1-2 were other transporters in the network. AtSUC1 has been shown expression in the FG of *Arabidopsis* [33]. ATPDIL1-2 had also high level of expression in FG. PDI proteins have key roles in a variety of cells, especially in cells involved in protein secretion and storage [48]. Interestingly, CAT1 was identified as a critical node in the network implying central possible roles for transporters during FG development.

The results of this study indicate numerous interactions of phytohormones in the network. According to the network and the findings of Zhang and O'Neil [49], auxin and ethylene coordinate in the development of the female and male gametophytes. Pagnussat *et al.* (2009) [50] showed that the patterning of FG depends on an asymmetric distribution of auxin during syncytial development, and its biosynthesis is location-specific.

ARF6 and ARF8 were found in the network based on the biological processes. ARF6 was also identified as a critical node in the network (Fig. 4B). ARF8 is expressed in FG, and its expression stops after fertilization [51]. Mutations in ARF8 and ARF6 result in female sterility [52]. This is consistent with the proposed role of ARF8 as a negative regulator of signal transduction in the ovules and during pistil growth before fruit initiation. The fold change for this gene was less than 0.66, but the protein plays an important role in ovules according to Goetz *et al.* (2006) [51]. Recently, Wang *et al.* (2010) [26] showed that ARF15 is expressed in antipodal cells. Auxin was linked to AT2G26740. Previously, Kiyosue *et al.* (1994) [53] found that AT2G26740 expression is induced by auxin. Another interesting interaction was between DD31 and ethylene. It was shown that DD31 is expressed specifically in synergids [3].

Another considerable finding was the presence of calmodulin and calcium binding proteins in the network, including AT5G43035, ANNAT1, and ANNAT4. Using forward genetics, Pagnussat *et al.* (2005) [2] showed that these two types of proteins are involved in the fusion of polar nuclei.

The interesting point is that distinct proteins belonging to certain families, such as those from the calmodulin and GGT families, are expressed in FG. It will be worthwhile to compare the promoters of all of the genes in a distinct family in future studies to find the answer of this phenomenon.

Surprisingly, there were some nodes related to cell death in the network. AMC6 is a member of the caspase family and induces apoptosis in yeast. FAH1 is a negative regulator of cell death. Cell death occurs in three types of cells during FG development, including three of four megaspores during megasporogenesis, antipodal cells during the late stage of FG development, and one of the synergid cells after entrance of the pollen tube. To date, a few mutation events affecting antipodal cells have been found [54]. Expression pattern analysis on these genes during FG development could be worthy to uncover more details.

Upregulated MYB98 was observed in the network. MYB98 plays specific roles in synergids and pollen tube guidance during FG development [1]. Although little is known about cell-cell communication, recently Chevalier *et al.* (2011) [55] pointed evidences for existence of cell-cell communication and signaling pathways during FG development. Interestingly, in the present network, signaling pathways and signal transduction were predicted as overrepresented GO terms (Table S6). Additionally, downregulated AHP2 (one of the six *Arabidopsis thaliana* histidine phosphotransfer proteins) involved in phosphorelay signaling system was predicted in the network. It appears that AHP2 plays a central role in signaling pathway during FG development [55]. The current network also shows interaction for DD39 with pollen development and ECA3. Besides expression in anthers, DD39 displays relatively strong expression in synergids and weak expression in egg cells [3]. DD39 encodes a polygalacturonase that plays a role in degrading the pollen mother cell wall during microspore development. It may have the same role in egg and synergid cells during fertilization. EOSTRE as another predicted protein in the network, controls synergid cell fate. In *eostre* mutant, one of two synergids displays polarity similar to egg cell [56]. Analyzing subnetworks of genes affecting both male and female gametophytes in the context of PI network in future studies can uncover more secrets about their functional relations and differential regulatory mechanisms during reproduction.

According to Johnston *et al.* (2007) [6], some genes downregulate in FG but upregulate in ovules of mutant *coatlique* lacking embryo sac. These genes such as AUXIN RESISTANT 2/3 (AXR2 and AXR3), SHORT INTERNODES (SHI), and SHOOT MERISTEMLESS (SHM1) were observed in the network. Interestingly SHI, SHM1 and AUX3 were critical proteins in the network based on biological processes.

GO analysis based on biological processes and molecular function showed similar results to previous reports [5]. Metabolic and cellular processes were the largest categories. Involvement of many different pathways implies complexity of this tiny organ. Signaling and transporters among molecular function categories are interesting unknown choices for future studies. The significantly overrepresented GO terms were also related to hydrolase activity. This is consistent with the analysis of the microarray data showing upregulation of different hydrolase family proteins, such as glycosyl hydrolase proteins AT5G24540 and AT5G24550. As reviewed by Yang *et al.* (2010) [57], a series of genes control cell cycle, embryo sac polarity, cell plate formation and cell specification during female gametogenesis. Consistent with these findings, our network has predicted all of significant biological processes (Table S6). The glycoside hydrolase family has role in many biological processes, such as control of hormone levels and the defense response [58], [59].

Recently Wuest *et al*. (2010) [5] reported cell type-specific expression profiles in the female gametophyte of *Arabidopsis* using laser-assisted microdissection and microarray techniques. Additionally, Johnston *et al.* (2007) [6] identified embryo sac expressed genes by genetic subtraction and microarray approaches. We looked for the identified genes by them are present in the network. Thirty six and 58 of FG identified genes by Wuest *et al.* (2010) [5] and Johnston *et al.* (2007) [6] were found in our network, respectively (Table S8). This analysis suggests that PI network can be extended by importing more FG expressed genes as preys. On the other hand, a number of selected upregulated and downregulated genes for construction of the networks could not build any edges (Table S9). This could be due to a lack of information about the function and biological process of many of these genes and their interactions which can be considered as a major factor for uncoverage of networks. Consequently, it is possible that many other proteins are not able to come into the network. It should be noted that the embryo sac and FG are arrested inside diploid tissues, and this makes mRNA isolation of the embryo sac cells difficult. Therefore, it is important to stress that the microarray data must be of the highest quality for constructing networks. Commonly, microarray data are verified by quantitative, reverse-transcriptase PCR; however, validations of this kind were not performed for all these data sets. Although, Wuest *et al.* (2010) [5] used laser-assisted microdissection to carry out more precise expression analysis, they might have lost due to hard amplification of low abundant transcripts. In the other hand, they used additional methods such as promoter:reporter gene fusions but it is really hard to apply for all genes. Therefore, it is possible that some unrelated proteins were falsely included in the network. The PI database also potentially contains false positives and false negatives which can affect the networks.

In this study, a variety of computational biology approaches were used to address and reduce false positives or false negatives errors in the network. Here, literature was used as references for predicted relations in the network and also microarray output data was applied into the network as another evidence for expression of the network members in FG. In this case, it should be noticed that some level of errors (false positives or false negatives) in the inferred network are accounted for the software and its false interpretation from cited database. However, many other important nodes were identified that the structure of the network can be disrupted by removing them. These nodes are important proteins involved in FG development. For this claim, we used evidences for verification of predicted protein interactions including different computational methods, experimental based data and microarray output data. However, further experiments should be carried out in order to obtain a general validation the obtained results as a limitation of the work.

This study presents PI network of FG development based on the transcriptome and interactome data. For the first time, the network demonstrates functional relations among a large number of previously identified genes. Analysis of the previously identified proteins predicts that they interact with novel proteins which had not been reported before. PI network also provides considerable clues for further functional analysis by identifying critical or essential proteins for FG development using topological analysis of the network. This study highlights the unique capability of network construction in prediction of novel undiscovered proteins. Furthermore, this study shows that a more clear image of complex phenomena such as FG development can be prepared based on network analysis and finding essential proteins/hubs in network trough computational biology methods. The discovered essential proteins will serve as a basis for future studies.

## Supporting Information

Table S1 **upregulated and downregulated genes selected based on available microarray datasets to infer the** ***Arabidopsis*** **FG protein interaction network**
(XLSX)

Table S2 **Summary of “Protein Relations” of FG protein interaction network based on “biological processes” (Primary network).**
(XLS)

Table S3 **Summary of “Protein Relations” of FG protein interaction network based on “biological processes” (subtracted network).**
(XLS)

Table S4 **Subtracted interaction network of MEA, DFR, JA, ABA, auxin, ethylene, SEC14, FAH, PR-4 based on biological processes**
(XLS)

Table S5 **Summary of “Protein Relations” of FG protein interaction network based on “Molecular function”**
(XLS)

Table S6 **Selected GO terms (“biological process” category) overrepresented in the female gametophyte interaction network, identified with the Biological Networks Gene ontology tool (BiNGO)**
(XLS)

Table S7 **Selected GO terms (“molecular function” category) overrepresented in the female gametophyte protein interaction network, identified with the Biological Networks Gene ontology tool (BiNGO)**
(XLS)

Table S8 **List of the genes identified previously by Johnston** ***et al.*** **(2007)** [**6**] **and Wuest** ***et al.*** **(2010)** [**5**] **which were predicted and present in the PI network of female gametophyte in this study**
(XLS)

Table S9 **List of the previously identified genes which could not build any edges in PI network of FG development.**
(XLS)
